# Incidence and Risk Factors of Obesity and Overweight in Kidney Transplant Recipients and Their Effect on Graft Outcome

**DOI:** 10.1155/joot/4990973

**Published:** 2025-11-06

**Authors:** Muhammad Abdul Mabood Khalil, Nihal Mohammed Sadagah, Hinda Hassan Khideer Mahmood, Abdulrahman Ibn Almuataz Wasfi Ezzi, Sultan Abdullah M. Alghamdi, Faisal Saud H. Alsulami, Ahmed Abdelahad Basha, Mohamed Abdelmonem Said Ahmed, Islam Nabil Sharabas, Hitham Abdallah Ahmed Abdallah Ragab, Aileen Jean Dela Cruz, Ghaleb Anas Aboalsamh, Salem H. Al-Qurashi

**Affiliations:** ^1^Centre of Renal Diseases and Transplantation, King Fahad Armed Forces Hospital, Al Kurnaysh Br Rd, Al Andalus, Jeddah 23311, Saudi Arabia; ^2^Department of Medicine, King Fahad Armed Forces Hospital, Al Kurnaysh Br Rd, Al Andalus, Jeddah 23311, Saudi Arabia

**Keywords:** kidney transplant recipient, obesity, overweight

## Abstract

**Background:**

Weight gain is common after kidney transplantation. This study assessed the incidence of overweight and obesity, identified the risk factors, and evaluated their impact on graft function and metabolic outcomes at 1 year.

**Methods:**

This retrospective observational study included 179 kidney transplant recipients at King Fahad Armed Forces Hospital, Jeddah, Saudi Arabia, between January 2020 and December 2023. The baseline and 12-month BMI were recorded. Associations with demographic, clinical, and metabolic variables, as well as graft function and complications, were analyzed. Logistic regression identified independent predictors of being overweight (BMI ≥ 25 kg/m^2^) and obese (BMI ≥ 30 kg/m^2^).

**Results:**

At baseline, 83 (46.4%) recipients had BMI < 25 kg/m^2^, 55 (30.7%) were overweight, and 41 (22.9%) were obese. At 12 months, the prevalence of obesity increased to 67 (37.4%), while the normal BMI category decreased to 55 (30.7%), with the overweight category remaining relatively stable at 57 (31.8%). The baseline BMI was the strongest predictor of overweight (OR 1.72, 95% CI 1.45–2.04) and obesity (OR 1.74, 95% CI 1.47–2.05) at 12 months. In sensitivity analysis excluding the baseline BMI, a family history of diabetes predicted obesity (OR 4.41, 95% CI 1.78–10.96). Obese patients had numerically higher creatinine and lower eGFR, but differences were not statistically significant. No differences were observed in CMV infection, prediabetes, new-onset diabetes after transplantation, acute coronary syndrome, or mortality. Overall, patient survival was 100% at 12 months.

**Conclusion:**

Posttransplant overweight and obesity are common. The baseline BMI and family history of diabetes are key predictors. The higher BMI is associated with early metabolic changes and trends toward lower graft function, highlighting the need for early monitoring and targeted interventions.

## 1. Introduction

Obesity and overweight are significantly high across the world. In 2022, an estimated 2.5 billion adults, or approximately 43% of the global population, were classified as overweight, with around 16% classified as obese—a figure that has doubled since 1990^1^. Obesity affects one in eight individuals and is associated with a substantial risk of noncommunicable diseases [1]. A higher body mass index (BMI) has been linked to 5 million deaths annually from noncommunicable diseases [2]. The situation in Saudi Arabia is even more alarming. The prevalence of obesity is around 35% [3], and mortality attributable to obesity is significantly higher than the global average (116.7 per 100,000 vs. 60 per 100,000) [4].

Weight gain following kidney transplantation is common, with most of the weight gain occurring within the first 6 months to 1-year posttransplant [5]. Several factors contribute to this phenomenon. Improved appetite resulting from better kidney function, reduced physical activity during recovery, corticosteroid-induced fat mobilization, and increased appetite are key contributors to posttransplant weight gain [6–8]. Weight gain posttransplant is linked to a higher prevalence of hypertension, dyslipidemia, and diabetes [9, 10]. These metabolic complications may, in turn, increase the risk of death-censored graft loss and death with a functioning graft, particularly in obese kidney transplant recipients [9, 11]. Although weight gain after kidney transplantation has been reported in several studies, most of these analyses are over a decade old and were mostly conducted in Western populations. Limited data are available from the Middle East, with one recent study from Saudi Arabia [8] examining posttransplant weight gain. Another study from Egypt was reported more than 2 decades ago [9]. However, these studies did not assess detailed BMI transitions, baseline predictors such as family history of diabetes, or associations with metabolic parameters and graft function. Therefore, contemporary, region-specific evidence is needed to better understand the risk factors and consequences of posttransplant weight gain.

Given the alarming prevalence of obesity and its associated mortality related to noncommunicable diseases in the general population in Saudi Arabia, this study was designed to investigate the incidence of overweight and obesity posttransplant over a 1-year follow-up period. The study also aims to identify factors associated with posttransplant overweight and obesity and their impact on transplant outcomes.

## 2. Methods

This retrospective observational study was conducted at King Fahad Armed Forces Hospital, Jeddah, Saudi Arabia, a tertiary care center with a capacity of 530 beds. The study focused on kidney transplant recipients who underwent transplantation between January 2020 and December 2023. Postoperatively, kidney transplant recipients are managed according to a standardized protocol, with long-term follow-up care provided in specialized transplant outpatient clinics. All patients with a baseline BMI < 35 kg/m^2^ were included, irrespective of retransplantation status or prior bariatric (sleeve gastrectomy) surgery. Exclusion criteria were pediatric recipients (< 14 years), multiorgan transplants, insufficient data to calculate BMI, and pregnancy during follow-up. This approach enabled the assessment of BMI trajectories and metabolic outcomes in a representative cohort, including individuals with retransplantation or prior bariatric interventions.

The primary objectives of this study were to determine the incidence of overweight and obesity among kidney transplant recipients, identify factors associated with posttransplant overweight/obesity, and assess their impact on renal allograft outcomes. Outcomes of interest included cytomegalovirus viremia, graft dysfunction, changes in low-density lipoprotein (LDL) cholesterol levels, posttransplant serum creatinine, development of prediabetes or new-onset diabetes mellitus, acute coronary syndrome (ACS), and mortality.

Baseline characteristics and complications were compared among BMI categories of < 25, 25–29.9, and ≥ 30 kg/m^2^. To know about prediabetes and new-onset diabetes mellitus after transplantation (NODAT), an analysis of subgroups of patients was performed after excluding diabetics and prediabetics. Patients were considered to have NODAT if they developed a fasting plasma glucose level ≥ 126 mg/dL (7.0 mmol/L) on at least two occasions, a random plasma glucose level ≥ 200 mg/dL (11.1 mmol/L) with classic symptoms of hyperglycemia, or an HbA1c level ≥ 6.5% after transplantation. In addition, the initiation of insulin or oral hypoglycemic therapy in previously nondiabetic patients was also considered diagnostic of NODAT. We also retrospectively looked into their past history of wound infection and urine leaks. BMI was calculated as weight in kilograms divided by the square of height in meters (kg/m^2^). Overweight was defined as a BMI ≥ 25–29.9 kg/m^2^, and obesity was defined as a BMI ≥ 30 kg/m^2^ [1]. To identify potential risk factors, patients were stratified into three cohorts based on BMI: those with BMI < 25, 25–29.9 and ≥ 30 kg/m^2^. Antithymocyte globulin (ATG) is predominantly used in induction, and basiliximab is used in selected cases. Maintenance immunosuppression includes triple therapy, including prednisolone, mycophenolate mofetil, and tacrolimus. No steroid minimization or avoidance protocols are used in our center. Kidney transplant recipients were identified through a centralized computerized medical records system, followed by a detailed review of patient charts. Data were recorded on a structured proforma. The baseline BMI was compared with measurements 1 year posttransplant to evaluate parameter changes. The baseline graft was defined as the creatinine or eGFR level at day 7 (the time of discharge). Comparative analyses were performed to assess the association of overweight/obesity with demographic and clinical variables and its impact on graft function and other outcomes, as explained.

### 2.1. Statistical Analysis

Data collected for this study included demographic information (age, gender, and relevant baseline characteristics), cause of ESRD, type of renal replacement therapy (hemodialysis vs. peritoneal dialysis), deceased donor status, transplant type, and retransplant status, duration on dialysis before transplantation (dialysis vintage), induction therapy, maintenance immunosuppressive medications, history of smoking, family history of diabetes, LDL cholesterol levels, baseline HbA1c, and graft function at baseline. Each patient's baseline BMI was recorded and compared with their BMI at 1 year posttransplant to assess changes over time.

Descriptive statistics were used to summarize baseline demographic and clinical data. Continuous quantitative variables were expressed as the mean, median, interquartile range (IQR), and standard deviation (SD). Categorical qualitative variables were summarized as frequencies and percentages. Pairwise comparisons evaluated changes in BMI categories between baseline and 1 year posttransplant. Differences between groups were assessed using chi-square tests for categorical variables and independent *t*-tests for continuous variables. For non-normally distributed continuous variables, the Kruskal–Wallis test was used. The Bowker test was applied to analyze overall changes in BMI distribution over time. Multivariable logistic regression analysis was performed to identify independent predictors of overweight and obesity at 1 year.

Statistical significance was determined using two-tailed tests, with a *p* value of less than 0.05 considered statistically significant. All statistical analyses were performed using Python running on the Jupyter Notebook environment.

## 3. Results

### 3.1. General Characteristics and Clinical and Laboratory Parameters of the Study Population

A total of 179 kidney transplant recipients with complete BMI data were analyzed. Median age was 42.0 years (IQR 31.0–57.0), with 112 males (62.60%) and 67 females (37.40%). Nonsmokers comprised 101 recipients (56.40%), and 78 (43.60%) were smokers. Living-related transplantation was the most common (165, 92.20%), followed by deceased donor (4, 2.20%) and living-unrelated (3, 1.70%) transplants. Retransplantation occurred in two recipients (1.10%). The majority of recipients had an unknown primary renal disease (53.10%), reflecting limitations in historical documentation or referral patterns. The remaining cases included diabetic kidney disease, glomerulonephritis, polycystic kidney disease, autoimmune, congenital, and hypertension-related causes. While this may limit detailed analysis by primary disease, the study still provides valuable insights into posttransplant weight gain and metabolic outcomes in a real-world cohort.

At baseline, 83 recipients (46.4%) had BMI < 25 kg/m^2^, 55 (30.7%) were overweight (25–29.9 kg/m^2^), and 41 (22.9%) were obese (≥ 30 kg/m^2^). At 6 months, 66 (36.9%) had BMI < 25 kg/m^2^, 61 (34.1%) were overweight, and 52 (29.1%) were obese. At 12 months, obesity increased to 67 (37.4%), with 57 (31.8%) overweight and 55 (30.7%) < 25 kg/m^2^. Median HbA1c rose from 5.1% (IQR 4.4–5.7) at baseline to 6.1% (IQR 5.3–8.7) at 12 months. Median LDL cholesterol increased from 2.3 mmol/L (IQR 1.9–3.0) to 2.7 mmol/L (IQR 2.3–3.5). Impaired fasting glucose (5.6–6.9 mmol/L) was present in 91 recipients (50.8%), and 42 (23.5%) required insulin therapy.

Median eGFR increased from 63.0 mL/min/1.73 m^2^ at 1 week to 84.0 mL/min/1.73 m^2^ at 12 months, with median creatinine decreasing from 123.0 to 92.5 μmol/L. Last recorded eGFR and creatinine were 86.0 mL/min/1.73 m^2^ (IQR 72.0–99.5) and 87.0 μmol/L (IQR 72.0–106.0), respectively.

Immunosuppression included ATG in 148 recipients (82.7%) and basiliximab in 31 (17.3%). All patients received tacrolimus, MMF, and prednisone during the first 6 months. Median tacrolimus trough after discharge was 9.0 ng/mL (IQR 8.0–9.0), with a mean first-month trough of 8.5 ng/mL (IQR 7.0–10.0). Posttransplant complications were uncommon: ACS occurred in 10 recipients (5.6%), wound infection in 1 (0.6%), and CMV infection in 2 (1.1%). No urinary leaks were reported. There were no deaths during follow-up.  Table 1 shows the general characteristics and clinical and laboratory parameters of the study population.

### 3.2. BMI at Baseline, 6 Months, and 1 Year and Changes Over 1 Year

**Figure 1:**
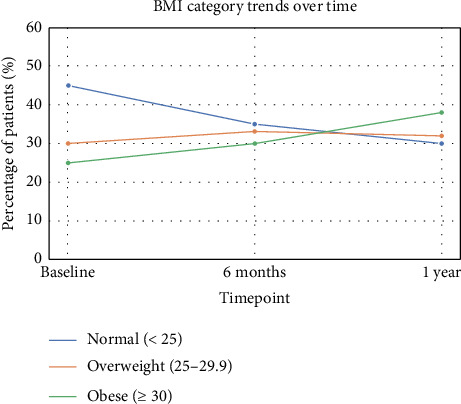
Trends in BMI categories over time in kidney transplant recipients. The proportion of patients with a normal BMI decreased from 46% at baseline to 31% at 12 months, while the proportion of patients with obesity increased from 23% to 38%. Overweight patients remained relatively stable at 30%.

During the first posttransplant year, BMI showed a progressive upward shift. At baseline, 83 patients (46.4%) were normal weight, 55 (30.7%) were overweight, and 41 (22.9%) were obese. By 6 months, the proportion of normal-weight patients declined to 66 (36.9%), while the proportions of overweight and obese patients increased to 61 (34.1%) and 52 (29.1%), respectively. At 12 months, only 55 patients (30.7%) remained normal weight, 57 (31.8%) were overweight, and 67 (37.4%) were obese. Among patients with normal BMI at baseline (*n* = 83), 27 (32.5%) became overweight and 6 (7.2%) progressed to obesity. Of those who were overweight at baseline (*n* = 55), 23 (41.8%) progressed to obesity, while 4 (7.3%) returned to a normal BMI. Among baseline obese patients (*n* = 41), the majority remained obese (38, 92.7%), with 2 (4.9%) becoming overweight and 1 (2.4%) returning to normal. Bowker's test confirmed a significant change in BMI distribution over time (chi-square = 113.7, df = 4, *p* < 0.0001), highlighting a notable trend of increasing BMI following kidney transplantation. Tables 2 and 3 show BMI categories at 6 and 12 months, respectively.  Table 4 shows changes in BMI from baseline to 12 months Figure 1 illustrates trends in BMI categories over the first year posttransplant. The proportion of patients with normal BMI decreased from 46.4% at baseline to 30.7% at 12 months, while the proportion of obese patients increased from 22.9% to 37.4%. Overweight patients remained relatively stable, with a change from 30.7% at baseline to 31.8% at 12 months. These trends indicate a progressive increase in BMI following kidney transplantation.

### 3.3. Predictors of BMI at 12 Months and Patient/Graft Outcomes

**Figure 2:**
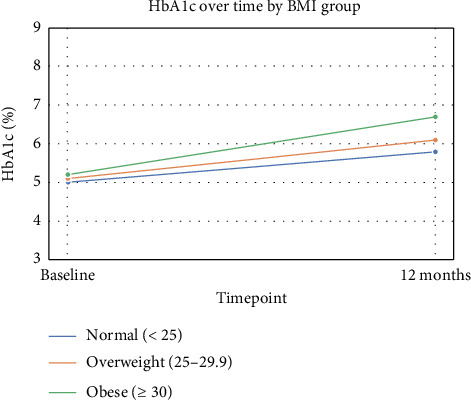
Trend in HbA1c among three groups over 1 year.

Baseline characteristics by the BMI category at the time of transplant are summarized in Table 5. Patients with a normal BMI (< 25) were younger (median age 35.0 [27.0–50.0] years) compared with overweight (52.0 [37.5–62.0] years) and obese patients (49.0 [37.0–60.0] years; *p* < 0.0001). The baseline BMI differed significantly across groups (normal: 21.4 [19.1–23.4], overweight: 27.0 [26.0–28.6], obese: 32.0 [31.1–33.6] kg/m^2^, *p* < 0.0001). A family history of diabetes was more common in overweight (29/55, 52.7%) and obese patients (24/41, 58.5%) than in normal-weight patients (24/83, 28.9%, *p*=0.0016). Induction therapy also varied, with ATG use higher in overweight (49/55, 89.1%) and obese patients (38/41, 92.7%) compared with normal weight (61/83, 73.5%, *p*=0.009). eGFR at 1 month was lower in obese patients (77.0 [66.0–92.0] mL/min/1.73 m^2^) than in normal (94.0 [79.0–114.0]) and overweight patients (88.0 [63.5–105.0], *p*=0.0073). After 1 year, the incidence of NODAT was 31.8% in the normal-weight group, 25% in the overweight group, and 32.5% in the obese group (*p*=0.7454, chi-square). Although not statistically significant, the highest incidence was observed among obese recipients. Similarly, new-onset prediabetes occurred in 15.9%, 30.6%, and 35.3% of normal-weight, overweight, and obese recipients, respectively (*p*=0.1222), again showing a higher frequency in obese patients. All groups demonstrated a gradual increase in HbA1c during the first year posttransplant, with higher mean values observed among obese recipients throughout the follow-up period (Figure 2). Gender, smoking status, donor type, retransplant, sleeve surgery, baseline HbA1c, LDL, and creatinine at 1 month showed no significant differences between BMI groups.

**Figure 3:**
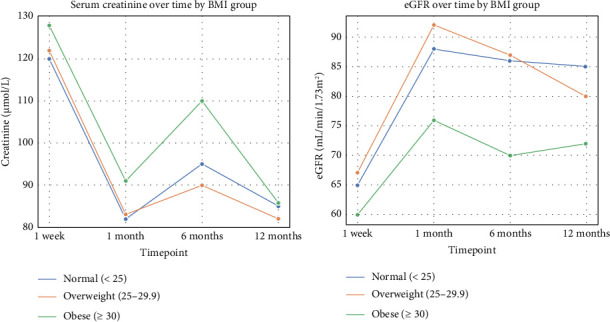
Trends in kidney function over time by the BMI group. Obese recipients had consistently higher creatinine and lower eGFR compared with normal BMI patients. eGFR peaked at 1 month in all groups and then declined, with obese patients showing the lowest values by 12 months. Patients with a normal BMI maintained the best graft function over time.

Characteristics by the BMI category at 12 months are shown in Table 6. The median age was higher in overweight (43.0 [31.0–58.0] years) and obese patients (48.0 [36.5–59.0] years) compared with normal-weight patients (35.0 [26.5–52.0] years, *p*=0.0040). The baseline BMI was significantly different across groups (normal: 20.4 [18.1–22.3], overweight: 25.3 [22.9–26.5], obese: 30.7 [27.5–32.7] kg/m^2^, *p* < 0.0001). A family history of diabetes was most common in obese patients (38/67, 56.7%) compared with overweight (22/57, 38.6%) and normal-weight (17/55, 30.9%, *p*=0.0118). Other variables, including gender, insulin use, smoking, donor type, ATG or basiliximab use, creatinine, eGFR, LDL, HbA1c, ACS, CMV infection, urinary leak, overall infection, and mortality, did not show significant differences across BMI groups.  Figure 3 illustrates trends in kidney function over time by the BMI group. Obese recipients consistently exhibited higher creatinine and lower eGFR compared with normal BMI patients. eGFR peaked at 1 month in all groups and then declined, with obese patients showing the lowest values by 12 months. In contrast, patients with normal BMI maintained the best graft function throughout the first posttransplant year; however, this difference was not statistically significant.

Multivariate logistic regression for factors is associated with BMI ≥ 25 and ≥ 30 at 12 months. In the main models (Table 7), the baseline BMI was the only independent predictor of BMI ≥ 25 (OR 1.72, 95% CI 1.45–2.04, *p* < 0.001). In the second model, after excluding the baseline, none of the variables emerged as significant predictors (Table 8). Additionally, the baseline BMI was also found to be an independent predictor of a BMI ≥ 30 (OR 1.74, 95% CI 1.47–2.05, *p* < 0.0001) as shown in Table 9. In sensitivity models excluding baseline BMI (Table 10), family history of diabetes was significantly associated with BMI ≥ 30 at 12 months (OR 4.41, 95% CI 1.78–10.96, *p*=0.0014), whereas age ≥ 50 years, smoking, baseline HbA1c, LDL, and eGFR showed no significant associations.

We observed that overall patient survival was 100% at 12 months, with no significant differences noted in ACS, CMV infection, urinary leak, or wound infection across BMI groups. Kidney graft function trends indicated that patients with normal BMI maintained the best function over time, while obese patients consistently exhibited higher creatinine levels and lower eGFR (Figure 3). However, these differences were not statistically significant.

## 4. Discussion

In this study of 179 kidney transplant recipients, we observed a significant and progressive increase in BMI during the first posttransplant year. The proportion of obese patients increased from 22.9% at baseline to 37.4% at 12 months, while the proportion of normal-weight recipients decreased from 46.4% to 30.7%, reflecting a notable trend of posttransplant weight gain. Interestingly, baseline obese patients largely remained obese (92.7%), whereas some normal-weight and overweight recipients shifted between BMI categories, demonstrating the dynamic nature of posttransplant weight changes. The baseline BMI was the strongest independent predictor of both overweight and obesity at 12 months, with a family history of diabetes also significantly associated with obesity in sensitivity analyses. Despite these weight changes, overall patient survival was 100% at 12 months, and posttransplant complications were uncommon. Obese recipients consistently exhibited higher creatinine and lower eGFR compared with normal BMI recipients although these differences were not statistically significant.

Previous studies from Saudi Arabia [8] (data 2016–2019, published 2022) and Egypt [9] (published 2004) have examined posttransplant weight gain. The Saudi study identified factors such as shorter dialysis duration, living donors, and baseline obesity, while the Egyptian study provided long-term follow-up. However, they neither captured detailed BMI trajectories during the first posttransplant year nor explored early predictors such as baseline BMI, family history of diabetes, and early eGFR changes. Our study complements this literature by tracking BMI at multiple time points within the first year and identifying early factors associated with weight gain, offering additional insights for posttransplant management.

Our study demonstrates a clear upward shift in BMI during the first year after transplantation. The proportion of patients with a normal BMI declined notably, while the prevalence of obesity increased. This reflects substantial posttransplant weight gain. Notably, recipients who were already overweight or obese at baseline were particularly vulnerable, with a large proportion progressing to higher BMI categories. In fact, nearly half of overweight individuals transitioned to obesity, and the vast majority of those who were obese at transplant remained so at 1 year. Even among patients with initially normal BMI, a considerable proportion developed overweight or obesity within the first year. These findings underscore that posttransplant weight gain is not confined to a specific subgroup but affects recipients across the BMI spectrum, highlighting the need for early recognition and targeted interventions. This trend likely reflects a combination of metabolic, hormonal, and behavioral factors. Corticosteroids can stimulate appetite, promote fat deposition, and increase insulin resistance [12, 13]. We noticed an increase in insulin use in overweight and obese patients although it was not statistically significant. Improvements in overall well-being and energy levels after transplantation may also lead to increased caloric intake and reduced catabolism, contributing to weight gain [14]. In line with this, the higher BMI was associated with early metabolic alterations. Median HbA1c values were modestly higher in overweight and obese recipients. In our study, both NODAT and prediabetes were numerically more frequent among obese recipients at 1 year posttransplant although the differences were not statistically significant. This trend may again reflect the greater degree of insulin resistance and metabolic stress commonly associated with higher body weight after transplantation.

Patients with baseline overweight or obesity are especially susceptible, and those with a family history of diabetes may have an additional predisposition to metabolic dysregulation. These mechanisms help explain the observed shift toward higher BMI categories and the persistence of obesity in a substantial proportion of recipients during the first posttransplant year, emphasizing the need for proactive monitoring and interventions to mitigate metabolic complications and support long-term transplant outcomes.

To explore factors associated with posttransplant overweight and obesity, we performed multivariate logistic regression analysis. The baseline BMI was the only independent predictor of BMI ≥ 25 at 1 year, highlighting the strong influence of pretransplant weight on posttransplant trajectories. A sensitivity analysis excluding the baseline BMI was performed to assess the potential contribution of other factors without the dominating effect of pretransplant weight. In this model, none of the other variables reached statistical significance. Age ≥ 50 years, smoking, and baseline HbA1c showed nonsignificant trends toward increased risk, whereas LDL cholesterol and early posttransplant eGFR showed no clear association. These results emphasize that although pretransplant BMI is the primary determinant of posttransplant weight gain, other potentially modifiable factors may still contribute and warrant attention in long-term patient management. Our findings, that baseline BMI is the strongest predictor of posttransplant overweight and obesity, are consistent with those of Schmid-Mohler et al. Schmid-Mohler et al. systematically reviewed risk factors for weight and body fat gain in the first year after kidney transplantation and identified baseline BMI, age, gender, smoking, and lifestyle factors among the most relevant predictors [15].

Next, we examined factors explicitly associated with obesity (BMI ≥ 30) at 1 year. As with overweight, the baseline BMI was the strongest independent predictor, emphasizing the dominant influence of pretransplant weight on posttransplant outcomes. In the sensitivity model excluding the baseline BMI, a family history of diabetes emerged as a significant predictor (OR 4.41, *p*=0.0014). This reflects that genetic or familial predisposition may contribute to the development of obesity after transplantation. While a family history of diabetes is a well-established risk factor for new-onset diabetes mellitus [16], no prior studies have directly linked it to obesity in kidney transplant recipients. However, in the general population, a family history of diabetes has been associated with obesity as reported in multiple studies [17–19]. Several mechanisms have been proposed. First, individuals with such a background often show an “obese phenotype” in adipose tissue. They have a reduced capacity to store fat in the subcutaneous compartment. As a result, excess fat tends to accumulate in ectopic sites such as the liver and skeletal muscle. This abnormal distribution of fat amplifies the metabolic consequences of obesity [20]. Second, shared genetic and epigenetic factors may predispose these individuals to altered insulin sensitivity, dysregulated energy metabolism, and a lower threshold for weight gain when exposed to environmental triggers [21]. In kidney transplant recipients, these inherited susceptibilities may be further compounded by transplant-specific exposures. Corticosteroids promote central adiposity, stimulate appetite, and impair adipocyte differentiation, all of which facilitate weight gain [22]. Calcineurin inhibitors, such as tacrolimus and cyclosporine, alter insulin sensitivity and pancreatic β-cell function, thereby exacerbating the metabolic burden of excess adiposity [23]. Additionally, rapid improvement in appetite following uremia reversal, reduced physical activity in the early posttransplant period, and lifestyle factors further increase the risk of obesity [24]. Given these biologically plausible pathways, it is reasonable to hypothesize that kidney transplant recipients with a family history of diabetes are more prone to posttransplant obesity. They may also face a higher risk of its complications. Further studies are needed to confirm this relationship. Such work could also help in developing tailored weight-management strategies for this high-risk group.

We observed numerical differences in graft function across BMI groups. Obese recipients tended to have higher creatinine and lower eGFR than normal BMI patients although these differences did not reach statistical significance. Our follow-up period was relatively short, and the long-term impact of these trends could not be determined. Normal BMI recipients generally maintained the best graft function over time. Since a family history of diabetes is associated with obesity in the general population, transplant recipients with such a history may be particularly prone to posttransplant weight gain and its metabolic consequences. Prior studies, including El-Agroudy et al. [9], have shown that posttransplant weight gain and obesity are associated with adverse graft and patient outcomes. Large registry data, such as the ANZDATA, further highlight the potential impact of obesity on delayed graft function, graft failure, and mortality [25]. Together, these observations underscore the importance of monitoring weight and metabolic risk and suggest the need for early, individualized interventions in high-risk kidney transplant recipients.

In our cohort, we did not find a significant difference in complications such as cytomegalovirus infection, prediabetes, NODAT, ACS, or mortality among kidney transplant recipients. The overall incidence of CMV infection was remarkably low at 1.1%, with no significant difference between recipients with BMI < 25 kg/m^2^, overweight, and obese recipients. This lower incidence could be attributed to the universal prophylaxis implemented in our cohort. While some studies have reported a lower incidence of CMV infection in kidney transplant recipients with weight gain [8, 26], our findings did not replicate these results. Further studies are needed to examine the association of weight gain and its relation with CMV viremia.

There are several limitations to our study. Our study is retrospective in nature, which may lead to selection bias. The study is also a single-center study, and its results may not be generalized to other transplant centers. One year of follow-up is short, and it may not catch up with the long-term consequences of being overweight or obese. Additionally, data on cold ischemia time were not included, which may influence graft outcomes and mortality.

Our study has several notable strengths. First, it provides essential insights into posttransplant overweight and obesity in kidney transplant recipients in Saudi Arabia, a population with limited data. Second, it identifies the baseline BMI and family history of diabetes as key risk factors for posttransplant obesity. Third, the cohort predominantly consisted of live donor transplants (97.8% of the total), resulting in a relatively homogeneous study population. This homogeneity reduces the confounding effect of donor-related factors, thereby enhancing the reliability of our findings.

## 5. Conclusion

Weight gain leading to overweight or obesity is a common problem after kidney transplantation. The baseline BMI is an important risk factor for developing posttransplant overweight and obesity. A family history of diabetes also predicts the risk of posttransplant obesity. Obese recipients tended to have higher creatinine and lower eGFR although these differences were not statistically significant in the short term. NODAT and prediabetes were numerically more frequent among obese recipients although the differences were not statistically significant. Median HbA1c also tended to be higher in obese recipients, suggesting a possible increased risk of posttransplant dysglycemia. Our findings address a gap in the regional literature on posttransplant weight gain. Future studies should explore preventive nutritional and lifestyle strategies tailored to kidney transplant recipients. There is also a need for more prospective studies to better understand the risk factors for posttransplant weight gain and its long-term consequences on graft function and patient outcomes.

## Figures and Tables

**Table 1:** General characteristics and clinical and laboratory parameters of the study population.

**Variable**	**Category**	**Count (N)**	**Percentage (%)**

	**Demographics (categorical variable)**		

Gender	Male	112	62.60
	Female	67	37.40
Smoking	Nonsmoker	101	56.40
	Smoker	78	43.60
ESRD cause	Unknown etiology	95	53.10
	Diabetic kidney disease	64	35.80
	Glomerulonephritis	9	5.02
	Adult polycystic kidney disease	4	2.23
	Autoimmune	3	1.70
	Congenital	3	1.70
	Hypertension	1	0.60
Deceased	No	169	94.40
	Yes	10	5.60
Transplant type	Living related	165	92.20
	Deceased donor	4	2.20
	Living emotionally related	3	1.70
Retransplant	No	177	98.90
	Yes	2	1.10
Renal replacement therapy	Hemodialysis	171	95.53
	Peritoneal dialysis	8	4.46%
	No	5	2.80
Sleeve surgery	No	177	98.90
	Yes	2	1.10
Baseline BMI (kg/m^2^)	< 25 (normal/underweight)	83	46.4
	25–29.9 (overweight)	55	30.7
	≥ 30 (obese)	41	22.9
BMI category at 6 months (kg/m^2^)	< 25 (normal/underweight)	66	36.9
	25–29.9 (overweight)	61	34.1
	≥ 30 (obese)	52	29.1
BMI category at 12 months (kg/m^2^)	≥ 30(obese)	67	37.4
	25–29.9 (overweight)	57	31.8
	< 25 (normal/underweight)	55	30.7

**Demographics (continuous variables)**			

**Variable**	**Median**	**IQR**	**Unit**

Recipient age	42.0	[31.0–57.0]	Years
Baseline HbA1c	5.1	[4.4–5.7]	%
Baseline LDL	2.3	[1.9–3.0]	mmol/L
LDL cholesterol at 12 months	2.7	[2.3–3.5]	mmol/L
HbA1c at 12 months	6.1	[5.3–8.7]	%

**Kidney function (continuous variables)**			

eGFR at 1 week	63.0	[39.0–93.5]	mL/min/1.73 m^2^
eGFR at 1 month	88.0	[70.5–107.5]	mL/min/1.73 m^2^
eGFR at 6 months	80.0	[65.0–94.5]	mL/min/1.73 m^2^
eGFR at 12 months	84.0	[66.2–100.5]	mL/min/1.73 m^2^
Last recorded eGFR	86.0	[72.0–99.5]	mL/min/1.73 m^2^
Creatinine at 1 week	123.0	[81.5–168.0]	µmol/L
Creatinine at 1 month	85.0	[71.5–102.5]	µmol/L
Creatinine at 6 months	95.0	[78.0–112.5]	µmol/L
Creatinine at 12 months	92.5	[73.0–109.0]	µmol/L
Last recorded creatinine	87.0	[72.0–106.0]	µmol/L

**Immunosuppression and complications (categorical variables)**			

**Variable**	**Category**	**Count (N)**	**Percentage (%)**

ATG	Yes	148	82.7
	No	31	17.3
Basiliximab	No	148	82.7
	Yes	31	17.3
Tacrolimus	Yes	179	100
Cyclosporine	No	179	100
MMF	Yes	179	100
Use of methylpred induction	Yes	179	100
Pred use first week	Yes	179	100
Pred use at 3 months	Yes	179	100
Pred use at 6 months	Yes	179	100
ACS	No	169	94.4
	Yes	10	5.6
Infection	No	178	99.4
	Yes	1	0.6
Urinary leak	No	179	100
CMV prophylaxis	Yes	179	100
CMV infection	No	177	98.9
	Yes	2	1.1

**Immunosuppression and complications (continuous variables)**			

**Variable**	**Median**	**IQR**	**Unit**

Tacrolimus mean trough after DC	9.0	8.0–9.0	ng/mL
Mean trough FK level of first month	8.5	7.0–10.0	ng/mL

**Metabolic outcomes (categorical variables)**			

**Variable**	**Category**	**Count (N)**	**Percentage (%)**

Impaired FBS (5.6–6.9)	Yes	91	50.8
	No	88	49.2
Insulin use	No	135	75.4
	Yes	42	23.5
Mortality	No	178	100.0

**Table 2:** BMI distribution at transplant, 6 months, and 1 year.

BMI category	At transplant (*n*, %)	At 6 months (*n*, %)	At 1 year (*n*, %)
Normal (< 25)	83 (46.4%)	66 (36.9%)	55 (30.7%)
Overweight (25–29.9)	55 (30.7%)	61 (34.1%)	57 (31.8%)
Obese (≥ 30)	41 (22.9%)	52 (29.1%)	67 (37.4%)

**Table 3:** Changes in BMI categories from baseline to 12 months posttransplant.

Baseline BMI	Stayed normal	Became overweight	Became obese
Normal (< 25) (*n* = 83)	50 (60.2%)	27 (32.5%)	6 (7.2%)
Overweight (25–29.9) (*n* = 55)	4 (7.3%)	28 (50.9%)	23 (41.8%)
Obese (≥ 30) (*n* = 41)	1 (2.4%)	2 (4.9%)	38 (92.7%)

**Table 4:** Changes in BMI categories from baseline to 12 months posttransplant (Bowker test).

Baseline BMI category	Status at 12 months	*n* (%)	*p* value (Bowker test)
Normal (< 25) (*n* = 83)	Stayed normal	50 (60.2%)	< 0.001
	Became overweight	27 (32.5%)	
	Became obese	6 (7.2%)	

Overweight (25–29.9) (*n* = 55)	Stayed overweight	28 (50.9%)	< 0.001
	Became obese	23 (41.8%)	
	Became normal	4 (7.3%)	

Obese (≥ 30) (*n* = 41)	Stayed obese	38 (92.7%)	Reference
	Became overweight	2 (4.9%)	
	Became normal	1 (2.4%)	

*Note:* Bowker's test confirmed a significant change in BMI distribution over time (chi-square = 113.7, df = 4, *p* < 0.0001).

**Table 5:** Baseline characteristics by the BMI group at transplant.

Variable	Normal (< 25)	Overweight (25–29.9)	Obese (≥ 30)	*p* value
Recipient age	35.0 [27.0–50.0]	52.0 [37.5–62.0]	49.0 [37.0–60.0]	< 0.0001 (Kruskal–Wallis)
Gender	32 (38.6%) females; 51 (61.4%) males	19 (34.5%) females; 36 (65.5%) males	16 (39.0%) females; 25 (61.0%) males	0.8673 (chi-square)
Baseline BMI (kg/m^2^)	21.4 [19.1–23.4]	27.0 [26.0–28.6]	32.0 [31.1–33.6]	< 0.0001 (Kruskal–Wallis)
Family history of diabetes	24 (28.9%) yes; 59 (71.1%) no	29 (52.7%) yes; 26 (47.3%) no	24 (58.5%) yes; 17 (41.5%) no	0.0016 (chi-square)
Smoking	53 (63.9%) nonsmoker; 30 (36.1%) smoker	26 (47.3%) nonsmoker; 29 (52.7%) smoker	22 (53.7%) nonsmoker; 19 (46.3%) smoker	0.145 (chi-square)
Donor type	2 (2.4%) deceased donor; 79 (96.3%) living related; 1 (1.2%) living emotionally related	2 (3.9%) deceased donor; 48 (94.1%) living related; 1 (2.0%) living emotionally related	0 (0.0%) deceased donor; 38 (97.4%) living related; 1 (2.6%) living emotionally related	0.774 (chi-square)
Cause of ESRD	71 (85.5%) unknown; 1 (1.2%) diabetic kidney disease; 4 (4.8%) glomerulonephritis; 2 (2.4%) autoimmune; 3 (3.6%) adult polycystic kidney disease; 2 (2.4%) congenital; 1 (1.2%) HTN	46 (83.6%) unknown; 2 (3.6%) diabetic kidney disease; 3 (5.5%) glomerulonephritis; 1 (1.8%) autoimmune; 1 (1.8%) PKD; 1 (1.8%) congenital; 1 (1.8%) HTN	38 (92.7%) unknown; 1 (2.4%) diabetic nephropathy; 2 (4.9%) GN	0.858 (chi-square)
Retransplant	81 (97.6%) no; 2 (2.4%) yes	55 (100.0%) no; 0 (0.0%) yes	41 (100.0%) no; 0 (0.0%) yes	0.310 (chi-square)
Sleeve surgery	81 (97.6%) no; 2 (2.4%) yes	55 (100.0%) no; 0 (0.0%) yes	41 (100.0%) no; 0 (0.0%) yes	0.310 (chi-square)
Induction therapy	61 (73.5%) ATG; 22 (26.5%) basiliximab	49 (89.1%) ATG; 6 (10.9%) basiliximab	38 (92.7%) ATG; 3 (7.3%) basiliximab	0.009 (chi-square)
Creatinine at 1 month (µmol/L)	82.0 [71.0–100.0]	84.0 [68.5–100.0]	91.0 [76.0–113.0]	0.2019 (Kruskal–Wallis)
eGFR at 1 month (mL/min/1.73 m^2^)	94.0 [79.0–114.0]	88.0 [63.5–105.0]	77.0 [66.0–92.0]	0.0073 (Kruskal–Wallis)
Baseline HbA1c (%)	5.0 [4.7–5.6]	5.1 [4.8–6.2]	5.2 [4.8–6.6]	0.2255 (Kruskal–Wallis)
Baseline LDL (mmol/L)	2.4 [2.0–3.0]	2.2 [1.7–2.9]	2.1 [1.6–2.6]	0.1817 (Kruskal–Wallis)

**Table 6:** Characteristics by the BMI group at 1 year posttransplant with statistical tests included.

Variable	Normal (< 25)	Overweight (25–29.9)	Obese (≥ 30)	*p* value
Recipient age	35.0 [26.5–52.0]	43.0 [31.0–58.0]	48.0 [36.5–59.0]	0.0040 (Kruskal–Wallis)
Gender	31 (56.4%) males; 24 (43.6%) females	39 (68.4%) males; 18 (31.6%) females	42 (62.7%) males; 25 (37.3%) females	0.4193 (chi-square)
Baseline BMI (kg/m^2^)	20.4 [18.1–22.3]	25.3 [22.9–26.5]	30.7 [27.5–32.7]	< 0.0001 (Kruskal–Wallis)
Family history of diabetes	17 (30.9%) yes; 38 (69.1%) no	22 (38.6%) yes; 35 (61.4%) no	38 (56.7%) yes; 29 (43.3%) no	0.0118 (chi-square)
Insulin use	10 (18.9%) yes; 43 (81.1%) no	12 (21.1%) yes; 45 (78.9%) no	20 (29.9%) yes; 47 (70.1%) no	0.3159 (chi-square)
Smoking	18 (32.7%) smoker; 37 (67.3%) nonsmoker	25 (43.9%) smoker; 32 (56.1%) nonsmoker	35 (52.2%) smoker; 32 (47.8%) nonsmoker	0.0963 (chi-square)
Donor type	51 (94.4%) living related; 0 (0.0%) living emotionally related; 2 (3.7%) deceased donor	55 (98.2%) living related; 1 (1.8%) living emotionally related; 0 (0.0%) deceased donor	59 (95.2%) living related; 2 (3.2%) living emotionally related; 1 (1.6%) deceased donor	0.2286 (chi-square)
ATG (yes/no)	42 (76.4%) yes; 13 (23.6%) no	50 (87.7%) yes; 7 (12.3%) n	56 (83.6%) yes; 11 (16.4%) n	0.2751 (chi-square)
Basiliximab (yes/no)	13 (23.6%) yes; 42 (76.4%) no	7 (12.3%) yes; 50 (87.7%) no	11 (16.4%) yes; 56 (83.6%) n	0.2751 (chi-square)
Creatinine at 12 months (µmol/L)	90.5 [64.8–107.5]	79.5 [65.5–104.0]	91.0 [73.8–112.0]	0.3878 (Kruskal–Wallis)
eGFR at 12 months (mL/min/1.73 m^2^)	86.0 [70.5–105.0]	87.5 [66.5–102.2]	73.0 [65.5–89.0]	0.2018 (Kruskal–Wallis)
LDL cholesterol at 12 months	2.7 [2.2–2.9]	2.9 [2.28–3.78]	2.9 [2.4–3.5]	0.3560 (Kruskal–Wallis)
HbA1c at 12 months	5.75 [5.2–7.55]	6.1 [5.3–8.7]	6.2 [5.6–8.7]	0.3037 (Kruskal–Wallis)
New-onset of diabetes after transplantation (NODAT)	14 (31.8%)	9 (25%)	11 (32.5%)	0.7454 (chi-square)
New prediabetes	7 (15.9%)	11 (30.6%)	12 (35.3%)	0.1222 (chi-square)
ACS	2 (3.6%) yes; 53 (96.4%) no	5 (8.8%) yes; 52 (91.2%) no	3 (4.5%) yes; 64 (95.5%) no	0.4384 (chi-square)
CMV infection	0 (0.0%) yes; 55 (100.0%) no	2 (3.5%) yes; 55 (96.5%) no	0 (0.0%) yes; 67 (100.0%) no	0.1148 (chi-square)
Wound infection	0 (0.0%) yes; 55 (100.0%) no	1 (1.8%) yes; 56 (98.2%) no	0 (0.0%) yes; 67 (100.0%) no	0.3409 (chi-square)
Urinary leak	0 (0.0%) yes; 55 (100.0%) no	0 (0.0%) yes; 57 (100.0%) no	0 (0.0%) yes; 67 (100.0%) no	1.0000 (chi-square)
Mortality	0 (0.0%) yes; 55 (100.0%) no	0 (0.0%) yes; 57 (100.0%) no	0 (0.0%) yes; 66 (100.0%) no	1.0000 (chi-square)

**Table 7:** Multivariate logistic regression for factors associated with BMI ≥ 25 at 1 year (model including baseline BMI).

Predictor	OR	95% CI	*p* value
Age ≥ 50 years	0.94	0.29–3.04	0.92
Family history of diabetes	0.36	0.08–1.52	0.16
Smoking	0.84	0.32–2.24	0.73
Baseline BMI (kg/m^2^)	1.72	1.45–2.04	< 0.001
Baseline HbA1c (%)	1.50	0.88–2.54	0.13
LDL at transplant (mmol/L)	1.00	0.60–1.67	0.99
eGFR at 1 month (mL/min/1.73 m^2^)	1.01	0.99–1.02	0.43

**Table 8:** Multivariate logistic regression for factors associated with BMI ≥ 25 at 1 year (without baseline BMI).

Predictor	OR	95% CI	*p* value
Age ≥ 50 years	1.70	0.75–3.85	0.21
Family history of diabetes	1.17	0.46–3.00	0.74
Smoking	1.65	0.82–3.29	0.16
Baseline HbA1c (%)	1.18	0.86–1.64	0.30
LDL at transplant (mmol/L)	1.04	0.76–1.43	0.80
eGFR at 1 month (mL/min/1.73 m^2^)	1.00	0.99–1.01	0.63

**Table 9:** Multivariate logistic regression for factors associated with BMI ≥ 30 at 1 year (model including baseline BMI).

Predictor	OR	95% CI	*p* value
Age ≥ 50 years	0.45	0.14–1.48	0.1888
Family history of diabetes (Yes)	3.16	0.82–12.12	0.0934
Smoking (yes)	1.85	0.72–4.80	0.2033
Baseline BMI (kg/m^2^)	**1.74**	**1.47–2.05**	**< 0.0001**
Baseline HbA1c (%)	0.82	0.56–1.20	0.2977
LDL at transplant (mmol/L)	1.47	0.88–2.43	0.1382
eGFR at 1 month	1.00	0.98–1.02	0.9513

*Note:* The bold values indicate significant predictor of obesity.

**Table 10:** Multivariate logistic regression for factors associated with BMI ≥ 30 at 1 year (model excluding baseline BMI).

Predictor	OR	95% CI	*p* value
Age ≥ 50 years	0.72	0.33–1.59	0.4180
Family history of diabetes (Yes)	**4.41**	**1.78–10.96**	**0.0014**
Smoking (yes)	1.59	0.82–3.07	0.1677
Baseline HbA1c (%)	0.85	0.65–1.11	0.2216
LDL at transplant (mmol/L)	1.19	0.88–1.61	0.2700
eGFR at 1 month	0.99	0.98–1.01	0.2732

*Note:* The bold values was significantly associated with obesity at 1 year.

## Data Availability

The data that support the findings of this study are available on request from the corresponding author. The data are not publicly available due to privacy or ethical restrictions.

## Nomenclature

ACS: Acute coronary syndrome
ATG: Antithymocyte globulin
ANZDATA: Australia and New Zealand Dialysis and Transplant Registry
BMI: Body mass index
CI: Confidence interval
CKD: Chronic kidney disease
CMV: Cytomegalovirus
CS: Corticosteroid
DF: Degrees of freedom
DM: Diabetes mellitus
eGFR: Estimated glomerular filtration rate
ESRD: End-stage renal disease
HbA1c: Glycated hemoglobin
IQR: Interquartile range
LDL: Low-density lipoprotein
MMF: Mycophenolate mofetil
NODAT: New-onset diabetes after transplantation
OR: Odds ratio
SD: Standard deviation
SPSS: Statistical Package for the Social Sciences
vs: Versus

## References

[1] World Health Organization Obesity and Overweight. http://www.who.int/news-room/fact-sheets/detail/obesity-and-overweight.

[2] Murray C. J. L., Aravkin A. Y., Zheng P. (2020). Global Burden of 87 Risk Factors in 204 Countries and Territories, 1990–2019: A Systematic Analysis for the Global Burden of Disease Study 2019. *The Lancet*.

[3] DeNicola E., Aburizaiza O. S., Siddique A., Khwaja H., Carpenter D. O. (2015). Obesity and Public Health in the Kingdom of Saudi Arabia. *Reviews on Environmental Health*.

[4] GBD 2017 Risk Factor Collaborators (2018). Global, Regional, and National Comparative Risk Assessment of 84 Behavioural, Environmental and Occupational, and Metabolic Risks or Clusters of Risks for 195 Countries and Territories, 1990–2017: A Systematic Analysis for the Global Burden of Disease Study 2017. *Lancet*.

[5] Elster E. A., Leeser D. B., Morrissette C. (2008). Obesity Following Kidney Transplantation and Steroid Avoidance Immunosuppression. *Clinical Transplantation*.

[6] Diaz J. M., Sainz Z., Oliver A. (2005). Post-Renal Transplantation Weight Gain: Its Causes and Its Consequences. *Transplantation Proceedings*.

[7] Zelle D., Corpeleijn E., Klaassen G., Schutte E., Navis G., Bakker S. (2016). Fear of Movement and Low Self-Efficacy are Important Barriers in Physical Activity After Renal Transplantation. *PLoS One*.

[8] Altheaby A., Alajlan N., Shaheen M. F. (2022). Weight Gain After Renal Transplant: Incidence, Risk Factors, and Outcomes. *PLoS One*.

[9] el Agroudy A. E., Wafa E. W., Gheith O. E., Shehab El-Dein A. B., Ghoneim M. A. (2004). Weight Gain After Renal Transplantation is a Risk Factor for Patient and Graft Outcomes. *Transplantation*.

[10] Johnson C. P., Gallagher-Lepak S., Zhu Y. R. (1993). Factors Influencing Weight Gain After Renal Transplantation. *Transplantation*.

[11] Ducloux D., Kazory A., Simula-Faivre D., Chalopin J. M. (2005). One-Year Post-Transplant Weight Gain is a Risk Factor for Graft Loss. *American Journal of Transplantation*.

[12] Moreau K., Desseix A., Germain C. (2021). Evolution of Body Composition Following Successful Kidney Transplantation is Strongly Influenced by Physical Activity: Results of the CORPOS Study. *BMC Nephrology*.

[13] Bang J. B., Oh C. K., Kim Y. S. (2020). Insulin Secretion and Insulin Resistance Trajectories over 1 Year After Kidney Transplantation: a Multicenter Prospective Cohort Study. *Endocrinol Metab (Seoul)*.

[14] Nolte Fong J. V., Moore L. W. (2018). Nutrition Trends in Kidney Transplant Recipients: The Importance of Dietary Monitoring and Need for Evidence-Based Recommendations. *Frontiers of Medicine*.

[15] Schmid-Mohler G., Huber L., Mueller T. F. (2022). Variable Selection for Assessing Risk Factors for Weight and Body Fat Gain During the First Year After Kidney Transplantation. *Progress in Transplantation*.

[16] Xia M., Yang H., Tong X., Xie H., Cui F., Shuang W. (2021). Risk Factors for New-Onset Diabetes Mellitus After Kidney Transplantation: A Systematic Review and Meta-Analysis. *Journal of Diabetes Investigation*.

[17] Cederberg H., Stančáková A., Kuusisto J., Laakso M., Smith U. (2015). Family History of Type 2 Diabetes Increases the Risk of Both Obesity and its Complications: Is Type 2 Diabetes a Disease of Inappropriate Lipid Storage?. *Journal of Internal Medicine*.

[18] The InterAct Consortium (2013). The Link Between Family History and Risk of Type 2 Diabetes is Not Explained by Anthropometric, Lifestyle or Genetic Risk Factors: The EPIC-Interact Study. *Diabetologia*.

[19] van’t Riet E., Dekker J. M., Sun Q., Nijpels G., Hu F. B., van Dam R. M. (2010). Role of Adiposity and Lifestyle in the Relationship Between Family History of Diabetes and 20-Year Incidence of Type 2 Diabetes in U.S. Women. *Diabetes Care*.

[20] Arner P., Arner E., Hammarstedt A., Smith U. (2011). Genetic Predisposition for Type 2 Diabetes, But Not for Overweight/Obesity, is Associated With a Restricted Adipogenesis. *PLoS One*.

[21] Long Y., Mao C., Liu S., Tao Y., Xiao D. (2024). Epigenetic Modifications in Obesity-Associated Diseases. *MedComm*.

[22] Vali A., Beaupère C., Loubaresse A. (2024). Effects of Glucocorticoids on Adipose Tissue Plasticity. *Annales d’Endocrinologie*.

[23] Diker Cohen T., Dotan I., Calvarysky B., Robenshtok E. (2025). Endocrine Effects of Long-Term Calcineurin Inhibitor Use in Solid Organ Transplant Recipients. *European Journal of Endocrinology*.

[24] Stoler S. T., Chan M., Chadban S. J. (2023). Nutrition in the Management of Kidney Transplant Recipients. *Journal of Renal Nutrition*.

[25] Shi B., Ying T., Xu J., Wyburn K., Laurence J., Chadban S. J. (2023). Obesity is Associated With Delayed Graft Function in Kidney Transplant Recipients: A Paired Kidney Analysis. *Transplant International*.

[26] Forte C. C., Pedrollo E. F., Nicoletto B. B. (2020). Risk Factors Associated With Weight Gain After Kidney Transplantation: A Cohort Study. *PLoS One*.

